# Ciliary Neurotrophic Factor Protects Striatal Neurons against Excitotoxicity by Enhancing Glial Glutamate Uptake

**DOI:** 10.1371/journal.pone.0008550

**Published:** 2010-01-01

**Authors:** Corinne Beurrier, Mathilde Faideau, Khaled-Ezaheir Bennouar, Carole Escartin, Lydia Kerkerian-Le Goff, Gilles Bonvento, Paolo Gubellini

**Affiliations:** 1 Institut de Biologie du Développement de Marseille-Luminy (IBDML), UMR6216 (Centre National de la Recherche Scientifique/Université de la Méditerranée), Marseille, France; 2 Commissariat à l'Energie Atomique, Institut d'Imagerie Biomédicale (I2BM), Molecular Imaging Research Center (MIRCen), Fontenay-aux-Roses, France; 3 Centre National de la Recherche Scientifique, Commissariat à l'Energie Atomique URA2210, Fontenay-aux-Roses, France; University of North Dakota, United States of America

## Abstract

Ciliary neurotrophic factor (CNTF) is a potent neuroprotective cytokine in different animal models of glutamate-induced excitotoxicity, although its action mechanisms are still poorly characterized. We tested the hypothesis that an increased function of glial glutamate transporters (GTs) could underlie CNTF-mediated neuroprotection. We show that neuronal loss induced by *in vivo* striatal injection of the excitotoxin quinolinic acid (QA) was significantly reduced (by ∼75%) in CNTF-treated animals. In striatal slices, acute QA application dramatically inhibited corticostriatal field potentials (FPs), whose recovery was significantly higher in CNTF rats compared to controls (∼40% vs. ∼7%), confirming an enhanced resistance to excitotoxicity. The GT inhibitor dl-threo-β-benzyloxyaspartate greatly reduced FP recovery in CNTF rats, supporting the role of GT in CNTF-mediated neuroprotection. Whole-cell patch-clamp recordings from striatal medium spiny neurons showed no alteration of basic properties of striatal glutamatergic transmission in CNTF animals, but the increased effect of a low-affinity competitive glutamate receptor antagonist (γ-d-glutamylglycine) also suggested an enhanced GT function. These data strongly support our hypothesis that CNTF is neuroprotective via an increased function of glial GTs, and further confirms the therapeutic potential of CNTF for the clinical treatment of progressive neurodegenerative diseases involving glutamate overflow.

## Introduction

Ciliary neurotrophic factor (CNTF) is a neurotrophic cytokine belonging to the interleukin-6 type family. In the CNS, CNTF is released by astrocytes and stimulates the survival of developing neurons. CNTF is also neuroprotective in various models of acute neuronal death and neurodegenerative diseases [1], and it has been proposed as a neuroprotective agent for Huntington's disease (HD) [2]. In HD, glutamate receptor-mediated excitotoxicity is involved in the preferential loss of striatal medium-sized spiny neurons (MSNs). Neurons expressing high levels of NMDA receptors are lost early from the striatum of individuals affected with HD, and injection of NMDA receptor agonists such as quinolinic acid (QA) into the striatum of rodents or non-human primates mimics the pattern of neuronal damage observed in HD [3], [4]. CNTF administration in the striatum protects MSNs against QA in rodents and primates [5]–[7]. A phase I clinical trial confirmed the safety of local brain administration of encapsulated cells genetically engineered to produce CNTF and reported a recovery of somatosensory evoked potentials in patient implanted with capsules releasing the largest amount of CNTF [8]. Despite these encouraging results, the mechanisms mediating CNTF neuroprotective effect are still unclear. The change in astrocyte phenotype triggered by CNTF in the adult brain suggests that this cytokine may have an indirect neuroprotective effect through activated astrocytes [9]–[11]. Indeed, we have recently shown that CNTF-activated astrocytes display marked phenotypic and molecular changes associated with an improved handling of extracellular glutamate in the rat striatum [12]. We suggested that such effect could be mediated by an increased function of astrocyte glutamate transporters (GTs), GLAST and GLT-1. These two GTs uptake the bulk of extracellular glutamate [13] and this function is crucial to prevent accumulation of glutamate to excitotoxic levels. Using lentivirus-mediated CNTF overexpression in the rat striatum, whole-cell patch-clamp and extracellular electrophysiological recordings on corticostriatal slices, we provide evidence that CNTF neuroprotective effects against QA are mediated through an enhanced glutamate uptake by activated astrocytes.

## Results

### CNTF Activates Astrocytes and Protects Striatal Neurons against QA Excitotoxicity *In Vivo* and *In Slices*

**Figure 1:**
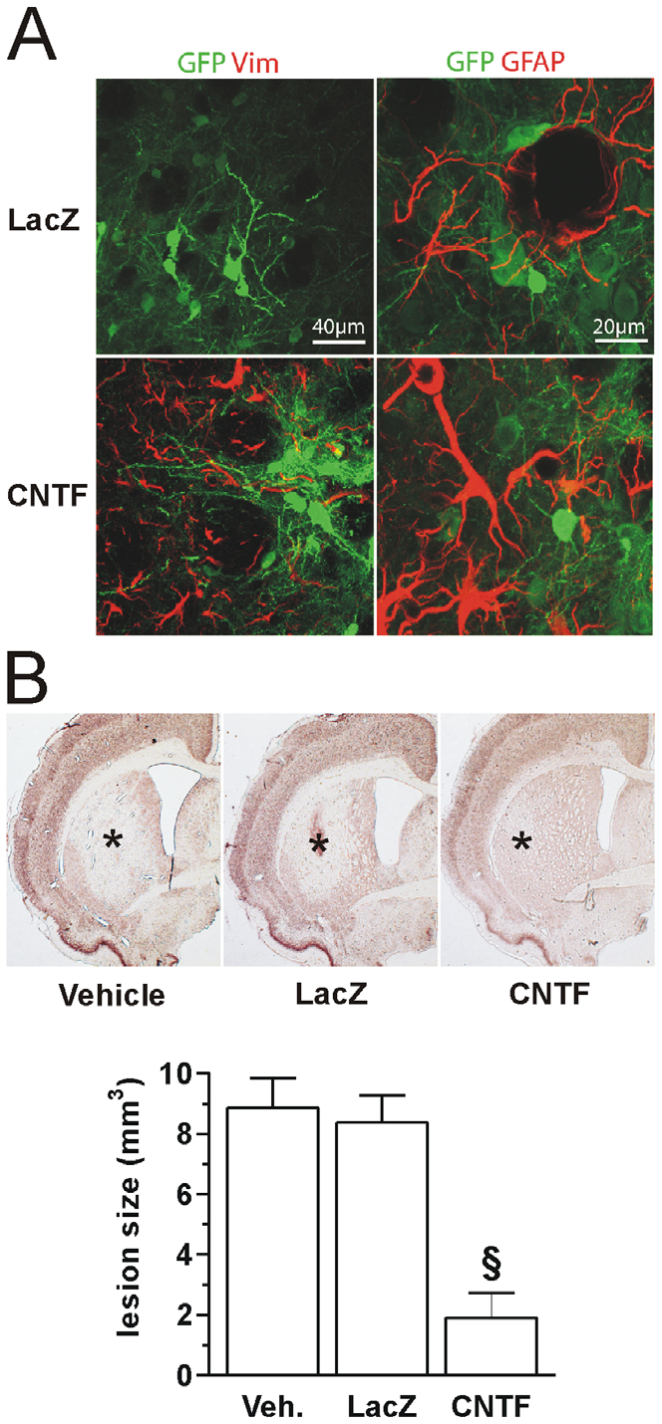
CNTF activates astrocytes and protects striatal neurons against QA excitotoxicity *in vivo*. **A:** CNTF activates astrocytes that re-express vimentin (red) and overexpress GFAP around GFP-positive MSNs neurons of the striatum. **B:** rats from Vehicle (Veh.), LacZ and CNTF groups were injected with 80 nmol QA and the lesion (*) volume was assessed 15 days later on NeuN-immunostained sections. CNTF significantly decreased lesion size (^§^p<0.001 vs. Veh. and LacZ groups, ANOVA and Scheffé's test).

We first confirmed that CNTF activated striatal astrocytes [12], [14]. We observed a marked re-expression of vimentin and the overexpression of GFAP (Fig. 1A) in CNTF rats. To evaluate CNTF neuroprotective effects against excitotoxicity, we used QA, which stimulates NMDA receptors and triggers glutamate outflow [15], [16]. QA was injected in the striatum of Vehicle, LacZ and CNTF rats and the volume of QA-induced lesions was measured on NeuN stained sections (N = 8-9/group) 2 weeks later. Lesion volume (Fig. 1B) was significantly smaller in the CNTF group (1.91±0.81 mm^3^) compared to both Vehicle (8.85±0.99 mm^3^) and LacZ (8.37±0.90 mm^3^).

**Figure 2:**
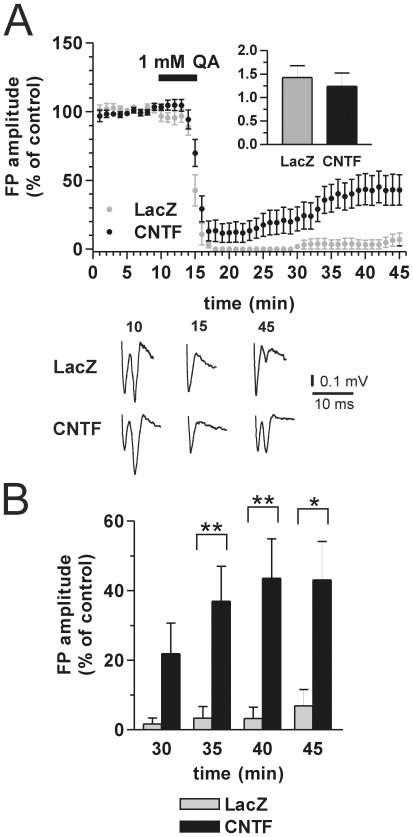
CNTF partially prevents QA-induced FP reduction in corticostriatal slices. **A:** time-course of the effect of QA on striatal FP amplitude in LacZ vs. CNTF slices. Note how FP loss in CNTF is attenuated and the recovery improved compared to LacZ. The inset shows that the input/output ratio (volley/FP) is similar in both groups. **B:** FP amplitude at different times in the two groups: FP recovery after QA washout is significantly improved in CNTF compared to LacZ rats (*p<0.05, **p<0.01, Mann-Whitney test).

We then tested the acute effect of QA application on FP recorded from corticostriatal slices. QA is known to induce a strong reduction in the amplitude of corticostriatal FP, followed by a partial recovery upon washout [17]–[19]. As shown in Fig. 2A, application of 1 mM QA for 5 min quickly depressed FP in slices from both LacZ and CNTF rats (N = 14 and 16, respectively). During QA washout, FP progressively and partially recovered in both groups. However, FP recovery was significantly improved in CNTF slices (Fig. 2B). For example, after 30 min QA washout, FP recovered only to 7±5% of the initial value in LacZ, but up to 43±11% in CNTF slices (p<0.05, Mann-Whitney test; Fig. 2B). To control that the improved FP recovery in CNTF rats was not due to a different basal synaptically-evoked glutamatergic activity, we measured the input/output ratio as the ratio of the presynaptic volley amplitude to the associated FP amplitude, before QA application. As shown in Fig. 2A inset, the input/output ratio was not significantly different between LacZ (1.43±0.25) and CNTF slices (1.24±0.28) (p>0.05, Mann-Whitney test). These data clearly show that CNTF overexpression in the striatum is neuroprotective against QA excitotoxicity.

### CNTF-Mediated Neuroprotection Is Not Due to Changes in Striatal Glutamatergic Transmission

**Figure 3:**
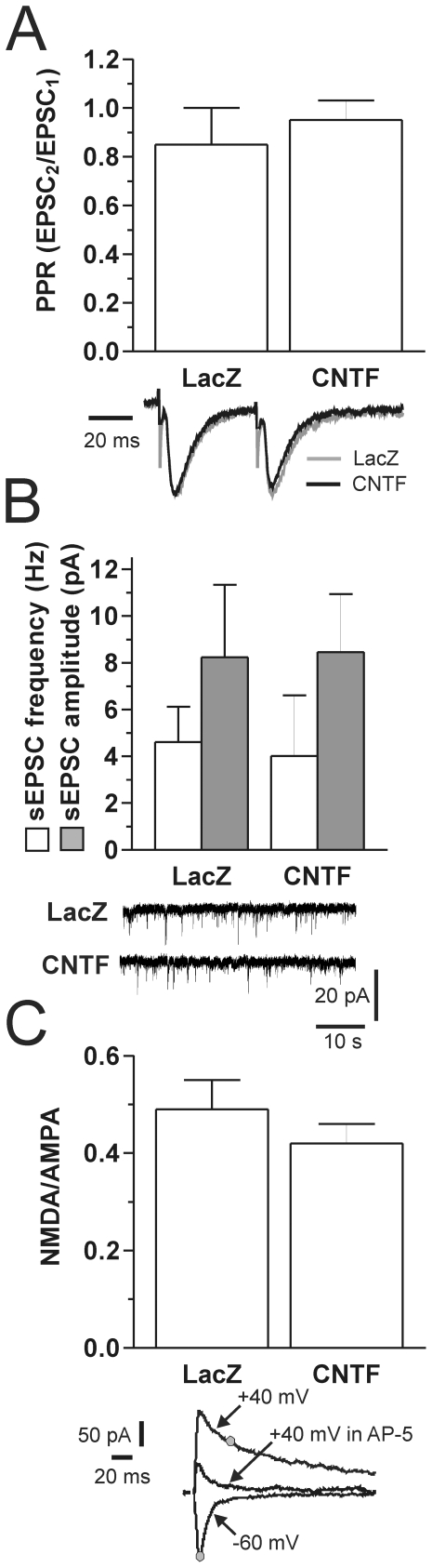
Properties of glutamatergic synaptic transmission recorded from striatal MSNs. No significant changes in PPR (**A**), average sEPSC frequency and amplitude (**B**), and NMDA/AMPA ratio (**C**) were observed between LacZ and CNTF slices. Traces: **A:** representative paired pulse-evoked EPSCs (normalized on the first black EPSC); **B:** examples of sEPSCs recordings; **C:** representative example of EPSCs recorded from a striatal MSNs (LacZ) showing where the AMPA and NMDA receptor-mediated component of the EPSC was measured (gray dots); note that after 50 ms (upper gray dot) the AMPA component is negligible, as shown by the trace in the presence of AP-5 (40 µM). HP in **A** and **B** was −60 mV.

Since the excitotoxic effect of QA is due to both glutamate release and NMDA receptor activation, we tested several parameters of basal glutamatergic transmission. Paired pulse ratio (PPR) of corticostriatal EPSCs (40 ms inter-EPSC interval; EPSC_2_/EPSC_1_) was not significantly different between LacZ (0.85±0.15; N = 10) and CNTF (0.95±0.08; N = 10) slices (p>0.05, Mann-Whitney test), indicating that glutamate release probability was not altered by CNTF (Fig. 3A). Frequency and amplitude of spontaneous EPSCs (sEPSCs) were also not changed by CNTF (Fig. 3B): average sEPSC frequency was 4.62±1.5 and 4.02±2.6 Hz, and average amplitude was 8.24±3.10 and 8.45±2.5 pA, respectively in LacZ and CNTF slices (N = 10 and p>0.05 for both frequency and amplitude, Mann-Whitney test). Finally, NMDA/AMPA ratio (Fig. 3C) was not significantly different between CNTF and LacZ slices (0.49±0.06, N = 31, and 0.42±0.04, N = 37, respectively, p>0.05, Student's t-test). These data suggest that the neuroprotective effects of CNTF are not due to major changes in glutamatergic transmission, glutamate release, and AMPA and NMDA receptor function.

### CNTF Overexpression Enhances Glial GT Function

**Figure 4:**
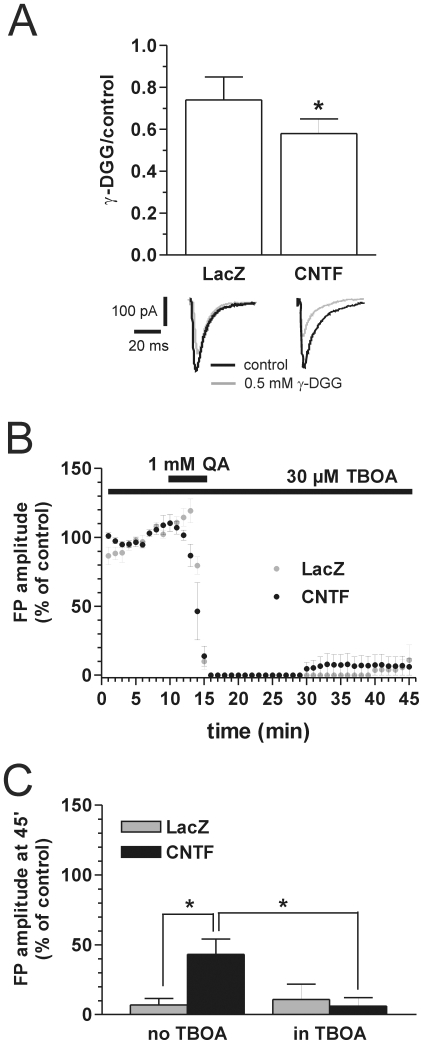
The neuroprotective effect of CNTF is mediated by GTs. **A:** γ-DGG has a significantly higher inhibitory effect in CNTF slices compared to LacZ: the histogram represents the ratio between EPSC amplitude in the presence of 0.5 mM γ-DGG and in control conditions (*p<0.05, Mann-Whitney test). Traces show representative EPSCs (HP = -60 mV). **B:** in the presence of the GTs inhibitor TBOA, there is little and similar FP recovery from QA excitotoxicity in both groups. **C:** summary of FP amplitude data at t = 45 min in the absence and in the presence of 30 µM TBOA (*p<0.05, Mann-Whitney test).

We then tested whether CNTF affected the function of GTs, as suggested by our microdialysis experiment showing improved handling of glutamate outflow in the rat brain [12]. We first used γ-d-glutamylglycine (γ-DGG), a low-affinity competitive glutamate receptor antagonist whose action is sensitive to the concentration and/or time-course of glutamate in the synaptic cleft [20]. The inhibitory effect of this drug is thus directly correlated to the efficacy of glutamate uptake: the more efficient the glutamate uptake, the stronger the γ-DGG effect [21]. Interestingly, the effect of γ-DGG (0.5 mM for 10 min) was significantly stronger in CNTF compared to LacZ slices, as shown in Fig. 4A. The ratio between EPSC amplitude in the presence of γ-DGG and in control conditions was 0.74±0.11 and 0.58±0.07 in LacZ (N = 6) and CNTF (N = 9) slices, respectively (p<0.05, Mann-Whitney test). These data indicate that CNTF overexpression reduces the relative concentration and/or time course of glutamate in the synaptic cleft, suggesting an enhanced glutamate uptake. To determine whether neuronal GTs were involved, we measured EPSC weighted decay-time constant (τ_w_), a parameter specifically modulated by EAAC1 in the striatum [22]. Interestingly, τ_w_ did not differ significantly between the two groups (LacZ: 9.69±0.99 ms, N = 9; CNTF: 13.43±1.67 ms, N = 6). Overall, these results suggest that glial, but not neuronal, GTs are potentiated by CNTF overexpression.

### CNTF Is Neuroprotective against QA by Increasing Glutamate Uptake

To address the involvement of GTs in CNTF neuroprotection, slices were incubated in 30 µM dl-threo-β-benzyloxyaspartate (TBOA), a wide spectrum GT inhibitor, before, during and after the application of QA. This dose of TBOA slightly increased FP amplitude in both groups, but such increase reached a steady state 15–20 min after drug application and was not significant (Fig. 4B). In the presence of 30 µM TBOA, the higher FP recovery in CNTF slices was no longer observed, because it was similar in the two groups. After 45 min of QA washout, FP amplitude was 11±10% and 6±6% of initial value in LacZ (N = 4) and CNTF (N = 6) slices, respectively; Fig. 4B, C. This result shows that GTs play a key role in the resilience of FP against QA excitotoxicity in CNTF slices.

## Discussion

In this study, we show that CNTF neuroprotective effects against glutamate excitotoxicity do not rely on a direct action on striatal MSNs and glutamate transmission, but are mediated by an enhanced glutamate uptake in activated astrocytes.

A number of studies have demonstrated that CNTF is a potent neuroprotective factor for striatal MSNs in animal HD models, including rodents and primates. CNTF was delivered either through minipumps [5], encapsulated genetically engineered cells [6], [7], [23], or by gene transfer with adenoviruses [24] and lentiviruses [25]. Here we show a marked neuroprotective effect of lentiviral-delivered CNTF against QA excitotoxicity *in vivo* (>75% neuronal rescue), and we also provide the first *in vitro* electrophysiological evidence that slices from CNTF animals are more resistant to QA. It has been shown that intrastriatal QA injection dramatically increases extracellular glutamate level [18], [26], and that this glutamate outflow plays a key role in QA-induced neurotoxicity through the activation of both ionotropic and metabotropic glutamate receptors [27]. Accordingly, we previously found that the neuroprotective effect of CNTF was associated with a marked reduction (>60%) of QA-induced increase in extracellular glutamate in the rat striatum [12]. Here we show that FP recovery during QA washout was significantly improved, suggesting that the massive glutamate outflow triggered by QA is more efficiently buffered in slices from CNTF-overexpressing rats [18].

CNTF binds to a tripartite receptor complex composed of the specific CNTF receptor alpha (CNTFRα) and two signal transducers, gp130 and leukemia inhibitory factor receptor [28]. The fact that CNTFRα is expressed at very low level in striatal neurons [29] and that CNTF has no neuroprotective activity in an almost pure neuronal culture system [30] suggests that this cytokine may act on other cells than neurons. Indeed, our patch-clamp recordings from striatal MSNs did not demonstrate any significant effect of CNTF at both pre- and postsynaptic levels (glutamate release probability, spontaneous activity and AMPA/NMDA receptor function). Expression of ionotropic glutamate receptor subunits NR2A, NR2B and GluR2, and of the vesicular glutamate transporter VGLUT1 was also previously shown not to be altered by CNTF [12]. Overall, these results suggest that CNTF does not modify striatal glutamatergic synaptic transmission.

The adult rat striatum expresses the glial glutamate transporters GLT-1 and GLAST, and the neuronal transporter EAAC1 [13], but astrocytes are responsible for the majority of extracellular glutamate removal. We have recently shown that glial GTs are involved in the handling of excess glutamate, for instance during tetanic synaptic activity, rather than modulating corticostriatal EPSCs [22]. Our present results using γ-DGG and TBOA suggest that CNTF enhances glutamate uptake and that such effect plays a significant role in the resistance of striatal neurons to QA-induced glutamate outflow. The fact that we did not observe significant changes in EPSC τ_w_ in CNTF slices supports the involvement of glial rather than neuronal GTs, since only the latter affect EPSC kinetic [22]. Indeed, CNTF increases the glycosylation level of GLAST and GLT-1, and redistributes them into raft microdomains in which glutamate uptake is more efficient [31] leading to a net increase in the glutamate buffering capacity, while no changes were observed for EAAC1 [12], [31].

Reduction of GLT-1 and/or GLAST expression and function has been reported in several neurodegenerative pathologies such as Alzheimer's disease [32], hippocampal sclerosis [33] and HD [34]. Interestingly, glutamate uptake inhibition in the rat striatum has been shown to trigger massive neuronal loss [35] mimicking excitotoxic HD models [3], and the expression of a mutant form of huntingtin decreases GLT-1 expression in transgenic HD mice [36] and in patients [34]. Altogether, these observations stress the role of GTs in HD and validate the concept of therapeutic approaches aimed at enhancing their function.

In conclusion, our data suggest that CNTF neuroprotective effects are mediated by astrocytes and support the notion that neuroprotection against glutamate excitotoxicity can be successfully achieved by increasing the capacity of glutamate buffering by astrocytes. Our findings further validate CNTF overexpression as a neuroprotective strategy for the treatment of HD and other neurological diseases characterized by glutamate-mediated neurodegeneration.

## Materials and Methods

### Injection of Lentiviruses in Rats

Three-month-old male Lewis rats (weight ∼300 g, IFFA Credo, France) were used in this study. All animal experimental procedures were carried out in strict accordance with the recommendations of the EEC (86/609/EEC) for care and use of laboratory animals, and conformed to the ethical guidelines of the French Ministry of Agriculture and Forests (Animal Health and Protection Veterinary Service). We used self-inactivated lentiviruses encoding either the human CNTF gene (lenti-CNTF) with the export sequence of immunoglobulin, or the β-galactosidase gene (lenti-LacZ) under the control of the mouse phosphoglycerate kinase 1 promoter [12], [25], [37]. Lentiviruses were diluted in vehicle [0.1 M phosphate buffer saline (PBS) with 1% bovine serum albumin] at a final concentration of 100 ng p24/ml. Rats were anesthetized with a mixture of ketamine (15 mg/kg) and xylazine (1.5 mg/kg). Suspensions of lenti-CNTF or lenti-LacZ were injected bilaterally into the striatum using a 10 µl Hamilton syringe (Reno, NV, USA) via a 28 gauge blunt needle (stereotaxic coordinates: AP +0.5 mm, L ±3.0 mm from bregma, V −4.5 mm from the dura, with tooth bar set at −3.3 mm). Rats received a total volume of 2 µl of lenti-CNTF (CNTF rats), lenti-LacZ (LacZ rats) or vehicle (Vehicle rats) per striatum at a rate of 0.2 µl/min. At the end of injection, the needle was left in place for 1 min before being slowly removed. The skin was sutured and rats were allowed to recover. CNTF effects were stable for several months without any sign of down-regulation.

### Injection of QA in Rats and Immunohistology

A solution of QA (80 mM in 0.1 M PBS) was prepared from a 180 mM stock solution containing 30 mg of QA dissolved in 900 µl PBS and 100 µl NaOH 1 N. CNTF, LacZ and Vehicle rats were anesthetized with a mixture of ketamine-xylazine, placed in a stereotaxic frame and injected with 1 µl of 80 mM QA using a blunt-tipped 25 gauge Hamilton syringe and the same stereotaxic coordinates as for lentiviral injections. Each injection was made over 1 min, and the needle was left in place 2 min before being slowly withdrawn. Two weeks later, rats were transcardially perfused with 4% paraformaldehyde under deep pentobarbital anesthesia. Brains were postfixed in 4% paraformaldehyde for 24 h and cryoprotected in a sucrose solution. Coronal brain sections (40 µm-thick) were cut on a freezing microtome, collected serially and processed for Neuronal Nuclei protein immunohistochemistry (NeuN, 1∶10,000, Chemicon, Billerica, MA, USA) with tyramine amplification [24]. Determination of the lesion volume was made on digitized sections (interspace 200 µm) by manually delineating the border of the lesion on each section. The total volume was calculated according to the Cavalieri method [38].

### Immunofluorescent Labeling

Staining was performed as described previously [12]. To identify MSNs, 2 animals were co-injected with a lentivirus encoding GFP (100 ng p24) and a lentivirus encoding CNTF or LacZ (200 ng p24). The antibodies used were directed against GFAP directly coupled to Cy3 (1∶500, Sigma, Saint-Louis, MO, USA) and against vimentin (1∶1000, Calbiochem, Nottingham, UK).

### Slice Preparation and Drugs

Brains were cut with a vibratome in coronal corticostriatal slices (250 µm) in ice-cold solution, whose composition was (in mM): 110 choline, 2.5 KCl, 1.25 NaH_2_PO_4_, 7 MgCl_2_, 0.5 CaCl_2_, 25 NaHCO_3_, 7 glucose, pH = 7.4 bubbled with O_2_/CO_2_ (95/5%). Slices were then stored in artificial cerebrospinal fluid (ACSF), whose composition was (in mM): 126 NaCl, 2.5 KCl, 1.2 MgCl_2_, 1.2 NaH_2_PO_4_, 2.4 CaCl_2_, 11 glucose, and 25 NaHCO_3_, pH = 7.4, gassed with O_2_/CO_2_, and containing 250 µM kynurenic acid and 1 mM sodium pyruvate. Recordings were performed at 35°C in standard ACSF solution (without kynurenic acid and sodium pyruvate) gassed with O_2_/CO_2_. Drugs were from Tocris-Cookson (Bristol, UK) and Sigma, and were dissolved in the perfusing ACSF at the desired final concentration.

### Electrophysiology and Data Analysis

Recordings were performed by AxoPatch 200B and Multiclamp 700B amplifiers with pClamp 10.2 software (Molecular Devices, Sunnyvale, CA, USA). Synaptic stimulation for triggering excitatory postsynaptic currents (EPSCs) and extracellular field potentials (FPs) was delivered by a bipolar electrode placed in the white matter between cortex and striatum. Whole-cell patch-clamp microelectrodes (4–5 MΩ) were filled with a CsCl-based solution, whose composition was (in mM): 140 CsCl, 10 NaCl, 0.1 CaCl_2_, 10 HEPES, 1 EGTA, 2 Mg-ATP and 0.5 Na-GTP, pH = 7.3. Striatal MSNs were visualized by infrared videomicroscopy before patching. Picrotoxin (50 µM) was always added to the bath solution to block GABA_A_ receptor-mediated synaptic transmission. Neurons showing ≥20% change in series resistance were discarded from the analysis. Extracellular recordings were performed using electrodes filled with 2 M NaCl (<2 MΩ). All recordings were obtained from the dorsal striatum close to the stimulating electrode, which delivered stimuli at 0.1 and 0.05 Hz for EPSCs and FPs, respectively. Data were analyzed offline by Clampfit 10.2 (Molecular Devices, Sunnyvale, CA, USA), Origin 7.5 (Originlab Corp., Northampton, MA, USA) and MiniAnalysis 6.0 (Synaptosoft, Decatur, GA, USA). EPSCs measurements were performed on samples of ten averaged traces, before and after each drug treatment. Amplitude was measured by averaging a 0.3 ms time interval centered on the maximum amplitude value (peak). Measurement of the AMPA and NMDA component of the EPSC for calculating NMDA/AMPA ratio was performed at peak for the AMPA component at a holding potential (HP) of −60 mV, and for the NMDA component at +40 mV and 50 ms after the stimulation artifact, when the AMPA component is negligible (see Fig. 3C). This protocol allows such measurements without using pharmacological blockers of ionotropic glutamate receptors, leaving the possibility to use the same MSN for other pharmacological tests [39]. FP amplitude was measured by averaging three consecutive sweeps. The mean basal FP amplitude was obtained by averaging a 10 min period before drug application. Statistical tests are specified case by case and data are presented as mean±SEM.
